# A Case of Immunoglobulin G4-Related Disease Presenting as a Parapharyngeal Mass

**DOI:** 10.7759/cureus.41764

**Published:** 2023-07-12

**Authors:** Arun Rathinam, Sushan Gupta, Mehwish Khan

**Affiliations:** 1 Internal Medicine, Carle Foundation Hospital, Urbana, USA; 2 Rheumatology, Carle Foundation Hospital, Urbana, USA

**Keywords:** igg4-related pseudotumor, igg4-related disease, parapharyngeal, igg4 disease, igg4

## Abstract

Immunoglobulin G4-related disease (IgG4-RD) is a fibroinflammatory condition characterized by tissue infiltration with lymphocytes and IgG4-secreting plasma cells. The presentation of IgG4-RD is heterogenous, making it difficult to diagnose. IgG4-RD presenting as a parapharyngeal mass is extremely rare. This report discusses the case of a 69-year-old African American female presenting with intermittent bilateral frontal headaches. Initial imaging revealed an ill-defined parapharyngeal mass encasing the left internal carotid artery and left internal jugular vein. Subsequent biopsy and immunohistochemistry showed a high concentration of IgG4-positive plasma cells with storiform fibrosis, despite normal serum IgG4 levels. The patient opted for conservative management. The localized parapharyngeal mass has remained stable over two years on annual imaging. This case report highlights that IgG4-RD can have varied and nonspecific presentations requiring high clinical suspicion to diagnose. Histopathology and IgG4 staining are vital to confirm the diagnosis of IgG4-RD, particularly in atypical cases not meeting the standard inclusion criteria.

## Introduction

Immunoglobulin G4-related disease (IgG4-RD) is an inflammatory condition characterized by tissue infiltration with lymphocytes and IgG4-secreting plasma cells and associated fibrosis. Initially described in 2001 in patients with sclerosing cholangitis [1], recent studies have described elevated IgG4 levels in a broad spectrum of diseases, including Mikulicz disease, autoimmune pancreatitis, hypophysitis, Reidel’s thyroiditis, Küttner tumor, interstitial pneumonitis, interstitial nephritis, retroperitoneal fibrosis, inflammatory aortic aneurysm, and aortitis, characterized as IgG4-RD spectrum [2]. Clinical manifestations depend on the organ system(s) involved and the severity of the disease [2]. IgG4-RD presenting as a parapharyngeal mass is extremely rare and can make diagnosing these cases exceptionally challenging. Here, we present the case of an elderly female with headaches who was diagnosed with an IgG4-related parapharyngeal mass.

## Case presentation

A 69-year-old African American female visited the emergency department (ED) multiple times over two months for intermittent bilateral temple headaches and retro-orbital pain, with associated photophobia, phonophobia, and hypersensitivity to odors. The headache worsened with bending, exertion, and coughing and often resolved spontaneously. She was treated for migraine during the ED visits and was referred to the neurology clinic for further workup. Her medical history was significant for migraine, diagnosed as a teenager, on daily maintenance topiramate and as-needed zolmitriptan pills. In the clinic, she endorsed experiencing worsening headaches, including severity, duration of individual headaches, and overall frequency for the past year, which did not improve with her current medications. The patient denied visual disturbances, nausea or vomiting, and loss of weight or appetite. The patient denied smoking, alcohol use, or other recreational drug use. Other medications included ascorbic acid, astragalus root, cholecalciferol, ginkgo, and multivitamin pills. The patient had two first-degree relatives (mother and sister) who died from brain aneurysms.

The patient’s vitals in the clinic were stable, with a blood pressure of 140 mmHg systolic and a heart rate of around 100 beats/minute. Her neurological examinations, including attention, concentration, memory, gait, visual fields, cranial nerves II-XII, power, tone, sensations (pain, light touch, vibration), deep tendon reflexes, proprioception, and coordination, were within normal limits. The rest of the physical examination was unremarkable.

Given her family history of brain aneurysms, magnetic resonance imaging (MRI) and magnetic resonance angiography (MRA) of the brain were done that showed a partially visualized mass in the left upper neck involving the adenoids, abutting and possibly involving the pterygoid muscles, encasing a portion of the left internal carotid artery, and abutting the longus coli muscles (measuring at least 2.2 cm × 4 cm × 4.7 cm). The MRA did not show any evidence of flow-limiting stenosis intracranially or any sizable intracranial aneurysms.

**Figure 1:**
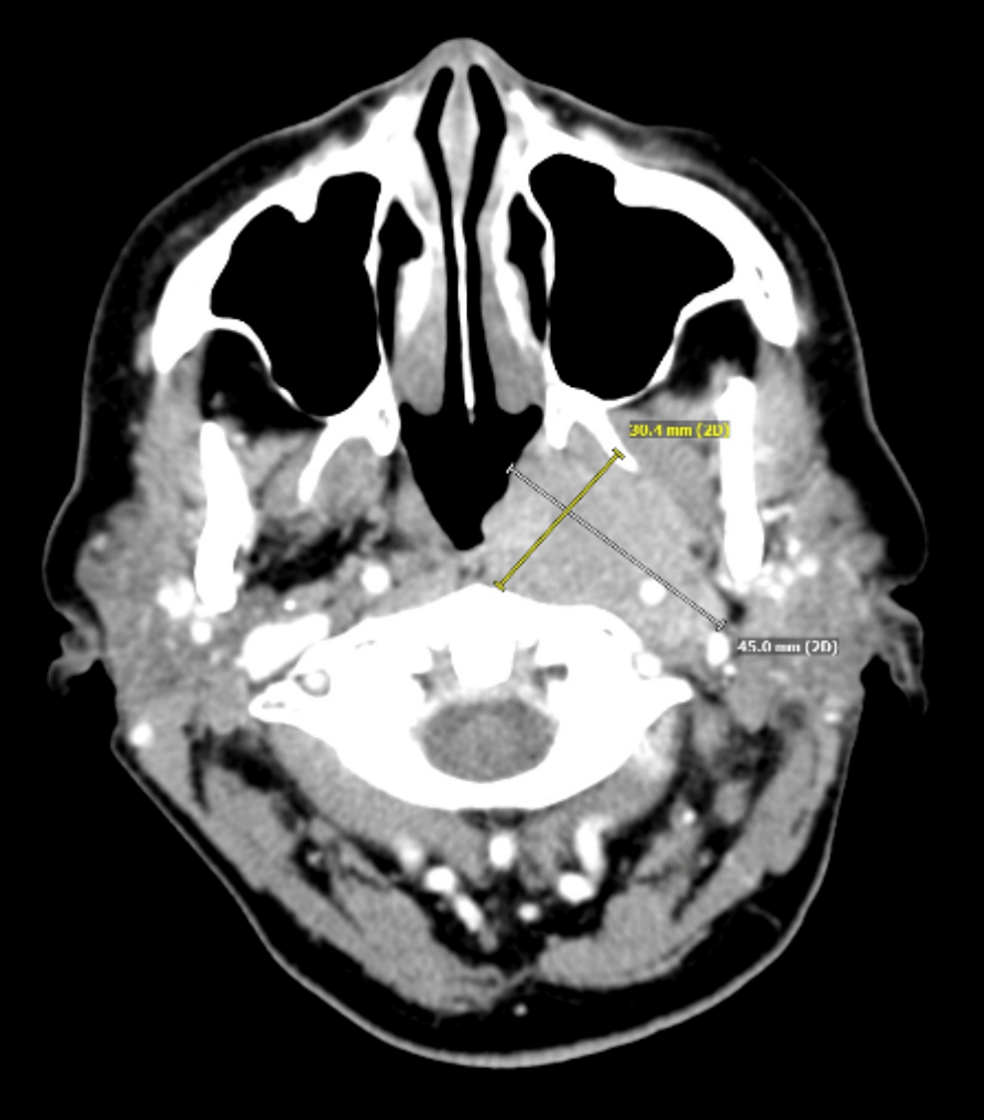
Computed tomography of the neck (axial view) demonstrating an ill-defined trans-spatial mass in the left upper neck below the base of the skull.

A subsequent computed tomography (CT) of the soft tissue of the neck showed an ill-defined contrast-enhancing trans-spatial mass in the left upper neck near the skull base (measuring approximately 4.5 cm × 3.0 cm × 4.1 cm) involving the posterior adenoids, torus tubarius, and left longus coli muscles. Posteriorly, it also encased the distal cervical left internal carotid artery and left internal jugular vein. Inferiorly, it extended to the superior margin of the left palatine tonsil. She also had a few level 2 left cervical lymph nodes that were asymmetric to the contralateral side but did not appear to be significantly enlarged (with the largest node measuring 1.1 cm × 0.8 cm) (Figure 1).

**Figure 2:**
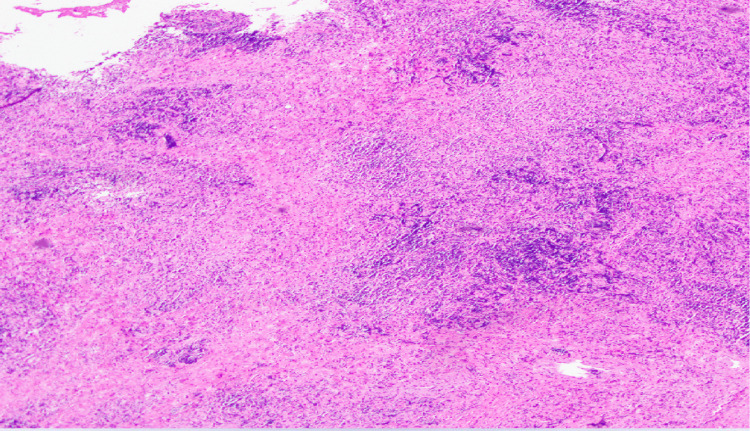
Histopathology showing storiform fibrosis with dense lymphoplasmacytic infiltrate.

**Figure 3:**
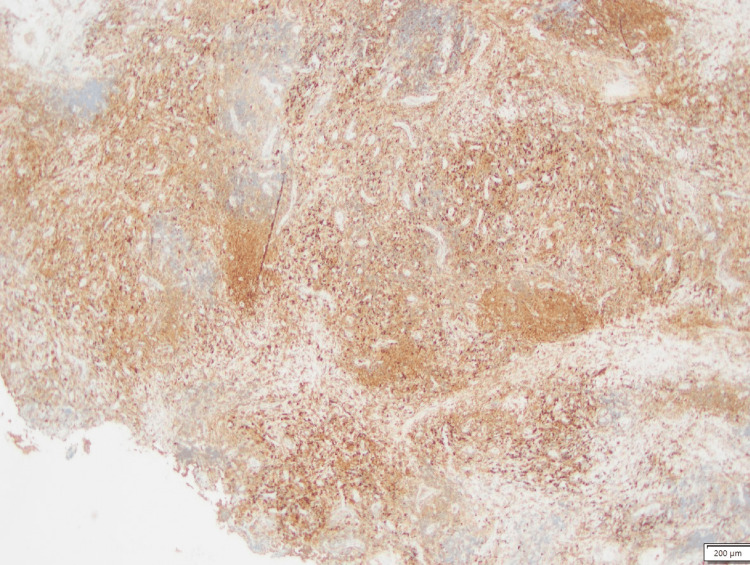
IgG4 staining of the section highlighting IgG4 cells.

The patient subsequently underwent an endoscopic nasopharyngeal biopsy of the parapharyngeal mass without any complications. Histopathology demonstrated glandular elements with fibrosis, dense lymphoplasmacytic inflammation with intermixed histiocytes, eosinophils, and periductal storiform fibrosis (Figure 2). No cellular atypia was noted. Immunohistochemistry showed that the lymphocytes were a mixture of CD3-positive T-cells and CD20-positive B-cells. CD138-positive plasma cells were polytypic for kappa and lambda immunoglobulin light chains. IgG4 staining revealed more than 100 IgG4-positive plasma cells per high-power field, and IgG4 cells were more than 40% of IgG-positive plasma cells (Figure 3).

The patient was referred to rheumatology for further management. The patient’s extensive autoimmune panel was negative, including serum complements C3 and C4, immunoglobulin E, TB Quantiferon, rheumatoid factor, anti-cyclic citrullinated peptide, and antinuclear antibody. Serum erythrocyte sedimentation rate and IgG4 levels were within normal limits. She also underwent a contrast-enhanced CT scan of the chest, abdomen, and pelvis, which ruled out any other system involvement. After discussing possible therapy options, the patient opted to hold off on initiating therapy with corticosteroids and any other systemic therapy due to concern for potential side effects. During the two years of follow-up, she continues to experience on-and-off headaches and swelling over the left side of her face. Periodic serum IgG4 levels have been within normal limits. The parapharyngeal mass has grossly remained unchanged in size and appearance on annual surveillance MRI.

## Discussion

IgG4-RD is a rare disease, and the exact prevalence of this condition is largely unknown [3]. Often presenting as lymphadenopathy with underlying autoimmune pancreatitis, it can involve multiorgan systems and have a heterogenous presentation [4]. Parapharyngeal involvement in IgG4-RD has been rarely described [5]. To the best of our review, there has been only one other documented case of IgG4-RD presenting as a parapharyngeal mass [6].

Generally categorized as an autoimmune condition, there has been no consensus on the exact pathogenesis of IgG4-RD. Many target antigens, including galectin-3, laminin-511, and annexin-A11, have been linked to the increased prevalence of IgG4-RD [7-9]. There is a growing theory that CD4+ T-cells are constantly activated by antigen-presenting B-cells [4]. This is supported by clinical remission in IgG4-RD that can be achieved with rituximab, an anti-CD20 monoclonal antibody, which causes B-cell depletion.

Previous studies have described IgG4-related diseases to involve the kidney, lung, gout bladder, pterygopalatine fossa, spleen, tongue, mediastinum, and submandibular gland leading to pseudotumor formation with different morphological presentations [10]. Our patient presented with a localized parapharyngeal mass surrounding the left internal carotid artery and left internal jugular vein. Interestingly, the mass has remained stable over two years since diagnosis despite the patient refusing systemic therapy.

Although the 2019 American College of Rheumatology/European League Against Rheumatism Classification Criteria for IgG4-RD delineates specific inclusion and exclusion criteria before undergoing biopsy and immunostaining to diagnose IgG4-RD, our patient did not meet one of the entry criteria (i.e., characteristic clinical or radiological involvement of a typical organ) [11]. Other differentials of an isolated parapharyngeal mass include sarcoidosis, malignancy, autoimmune vasculitis, and granulomatous infections (e.g., tuberculosis), which often need a biopsy to exclude. Our patient’s biopsy showed dense lymphoplasmacytic infiltrate with storiform fibrosis plus immunohistochemistry with IgG4 stain to highlight the IgG4+ cells and tissue IgG4:IgG ratio >40%, confirming the diagnosis of IgG4-RD [12]. Serum IgG4 levels should be tested in all patients suspected of having IgG4-RD, although normal serum levels cannot exclude IgG4-RD, as in our patient [13].

Based on the current consensus recommendations [14], glucocorticoids are the first line of treatment to induce remission in all patients with active, untreated, and symptomatic diseases. Watchful monitoring of asymptomatic lymphadenopathy or mild submandibular gland enlargement may be appropriate in select cases; however, the risk of progressing to irreversible organ damage exists [14]. Rituximab, a chimeric monoclonal immunoglobulin against the CD-20 receptor, is used in glucocorticoid-resistant cases or if patients cannot tolerate steroids. Initiation of therapy can lead to a rapid decline in serum IgG4 levels and rapid clinical improvement [15]. Immunomodulatory agents such as mycophenolate and azathioprine can also be used when rituximab is unavailable [16]. Depending on the anatomical regions and nearby structures affected, surgical resection or debulking may also be considered an option for highly fibrotic lesions [17]. A steroid-sparing maintenance therapy follows initial induction therapy and has been shown to reduce the risk of relapse [17]. The duration of therapy, however, remains controversial. Our patient denied medical treatment for the IgG4 disease and has not shown signs of progression for two years. However, despite stability, patients with IgG4 disease have an increased risk of developing malignancy throughout their lives [18].

## Conclusions

IgG4-RD can have a nonspecific and heterogenous presentation making the initial diagnosis challenging. Isolated parapharyngeal presentation is rare and often needs histopathology and IgG4 staining to confirm the diagnosis. Physicians should remain cognizant irrespective of the serum IgG4 levels.
